# Short Toxin-like Proteins Attack the Defense Line of Innate Immunity

**DOI:** 10.3390/toxins5071314

**Published:** 2013-07-23

**Authors:** Yitshak Tirosh, Dan Ofer, Tsiona Eliyahu, Michal Linial

**Affiliations:** Department of Biological Chemistry, Silberman Institute of Life Sciences, The Hebrew University of Jerusalem, Jerusalem 91904, Israel; E-Mails: yitshak.tirosh@mail.huji.ac.il (Y.T.); ddofer@gmail.com (D.O.); tsionae@cc.huji.ac.il (T.E.)

**Keywords:** neurotoxin, protein families, disulfide bonds, antimicrobial peptide, ion channel inhibitor, ClanTox, complete proteome, comparative proteomics, functional annotation

## Abstract

ClanTox (classifier of animal toxins) was developed for identifying toxin-like candidates from complete proteomes. Searching mammalian proteomes for short toxin-like proteins (coined TOLIPs) revealed a number of overlooked secreted short proteins with an abundance of cysteines throughout their sequences. We applied bioinformatics and data-mining methods to infer the function of several top predicted candidates. We focused on cysteine-rich peptides that adopt the fold of the three-finger proteins (TFPs). We identified a cluster of duplicated genes that share a structural similarity with elapid neurotoxins, such as α-bungarotoxin. In the murine proteome, there are about 60 such proteins that belong to the Ly6/uPAR family. These proteins are secreted or anchored to the cell membrane. Ly6/uPAR proteins are associated with a rich repertoire of functions, including binding to receptors and adhesion. Ly6/uPAR proteins modulate cell signaling in the context of brain functions and cells of the innate immune system. We postulate that TOLIPs, as modulators of cell signaling, may be associated with pathologies and cellular imbalance. We show that proteins of the Ly6/uPAR family are associated with cancer diagnosis and malfunction of the immune system.

## 1. Introduction

Short proteins are underrepresented among the proteomes of fully sequenced genomes. Currently, proteins that are <100 amino acids represent only 1.1% of the protein space. The low representation of short proteins reflects a failure of genome annotation prediction tools [1]. Furthermore, even sensitive large-scale technology, such as mass spectrometry (MS), provides only partial coverage of short proteins, mainly due to the abundance of post-translational modifications [2]. In recent years, with the explosion of next generation sequencing methods, many more transcripts have been identified, including non-coding RNAs [3] and numerous short overlooked transcripts. Still, validating short proteins as biologically active by mining genomics and proteomics sequences remains a challenging task.

Animal toxins and other short proteins share compact, cysteine rich scaffolds [4]. Recently, an increased number of proteins that resemble animal-toxins had been identified in non-venomous contexts [5]. These proteins often act as endogenous cell modulators [6]. They include pore-forming proteins, proteases and their inhibitors, cell antigens and growth factors [7,8].

In this study, we focus on the “three-fingered proteins” (TFPs) superfamily [9]. The term TFP is assigned to a fold composed of three adjacent loops emerging from a compact hydrophobic structure stabilized by 3–4 disulfide bonds. TFPs specify active components from elapids’ venoms. The first prototypes of TFP to be solved by X-ray crystallography was the α-neurotoxin from Philippine sea snake [10] and Cobra [11]. It was generalized that a typical TFP protein is a secreted polypeptide of 60–100 amino acids chains. This fold is often called “toxin fold”, due to the large number of elapid toxins that share it. The hallmark of such a fold is the five β-strands encompassing the three fingers. TFPs are identified in sporadic metazoan species along the phylogenetic tree, such as the sea urchin (*Hemicentrotus pulcherrimus*), tunicate (*Ciona intestinalis*), terrestrial nematode (*Caenorhabditis elegans*), fruit fly (*Drosophila melanogaster*) and mammals. Upon binding to the target protein, they act as modulators of cell surface receptors and may alter membrane signaling pathways. TFPs often act as ligands of the nicotinic (nAChR) or the muscarinic (mAChR) acetylcholine receptors. Other studied examples include the snake toxin fasciculin, which inhibits acetylcholinesterase, and the calciseptins that block the L-type calcium channels. In addition to their role in modulating ion channels and receptors, some TFPs act through pore formation or by competing with cell-adhesion processes [12].

Several predictors were developed for identifying short secreted toxin-like proteins from animals [13,14]. ConoServer is a unified resource for hundreds of peptides in the venom of marine snails of the genus, *Conus* [15]. Based on the discovery of cellular proteins that resemble toxins’ folds, it was proposed that a strong evolutionary relationship between animal toxins and ancestral cysteine cross-linked proteins exists [12,16,17]. Striking examples for snake α-neurotoxin-like proteins were identified in both the brain [18] and skin [19] from rodents and humans.

ClanTox (classifier of animal toxins) is a machine-learning based classifier for ranking protein sequences according to their toxin-like properties. ClanTox provides a characterization for proteins, which are mostly uncharacterized [6]. The short (<120 amino acids), secreted proteins that share toxin-like compact structures are collectively called toxin-like proteins (TOLIPs). The term TOLIP is used to describe proteins with features that are characteristic to animal toxins. Importantly, the expression or the function of TOLIPs is not associated with a venom gland or poisonous function. The dominating features of TOLIPs are an abundance of spaced cysteine residues, a high frequency of charge residues, a signal peptide for secretion and a compact structure. Importantly, TOLIPs are endogenous animals’ proteins that are not restricted to poisonous animals.

We have identified novel TOLIPs in the honeybee brain [6,20] and additional ones in viruses and rodents [5]. One of these proteins, OCLP1 (Omega-Conotoxin-Like Protein-1), is a 74 amino acid residue sequence that possesses a signal peptide. OCLP1 has a substantial similarity to a toxin from assassin bug that functions as a voltage-gated Ca^2+^ channel blocker. The impact of OCLP on the neuronal activity of the honeybee brain is not yet known.

The goal of this research is to fill the gap in knowledge on short proteins by describing the mammalian TOLIPs that belong to the Ly6/uPAR superfamily (acronyms for Lymphocyte Antigen 6 and Urokinase Receptor). The predicted functions of these TOLIPs are mostly unknown. However, some members were associated with the male reproduction system. For example, the SP-10 (also known as ACRV1 for acrosomal vesicle protein 1) is involved with binding of the sperm to the zona pellucida during fertilization [21]. Monoclonal SP-10 antibodies were found to inhibit sperm penetration [22]. Thus, SP-10 was suggested as a contraceptive immunogen.

We start with a brief description of the protocol for identifying TOLIPs from human and mouse transcriptomes. We summarize by showing that many of the Ly6/uPAR proteins in humans are associated with a repertoire of pathologies and diseases in the context of cancer diagnosis and the innate immune system. We conclude that different components of the innate immune system are the preferred sites for modulation by toxin-like proteins.

## 2. Results and Discussion

### 2.1. Discovering Natural Endogenous *α*-Neurotoxins in Mouse

Identifying short toxin-like proteins by applying homology search methods have mostly failed, due to the low sequence similarity and the broad functional diversity. To this end, we applied the computational classifier, called ClanTox [23]. The classifier was developed to take advantage of the shared features, such as the abundance of disulfide bridges and the conserved sites for post-translational modifications [24].

Briefly, our classifier, ClanTox, had been trained on known ion-channel inhibitors that are short and are signified by compact 3D structures. ClanTox ranks sequence candidates from mammals (mostly mouse and human) according to their resemblance to animal toxins from venomous animals (e.g., snakes, scorpions). A dominant feature for the classifier is the presence of numerous, spaced cysteine residues. Additionally, global properties of the sequence, such as the polarity of amino acids along the sequence, turned out to be discriminative. ClanTox identified previously overlooked TOLIPs [20].

**Figure 1:**
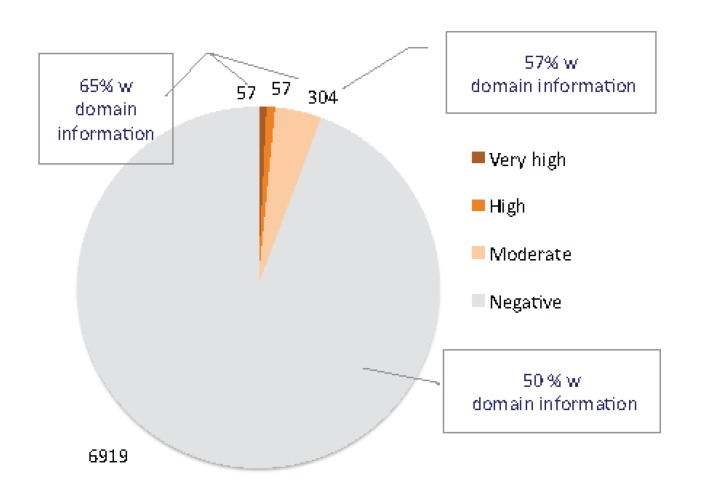
Classifier of animal toxins (ClanTox) results from mouse short proteome. Using 7337 short sequences as input to ClanTox resulted in 114 sequences (1.5%) that were scored as very high/high confidence levels. The dominant group from the high scored positive predictions are the β-defensins (beta defensin/neutrophil defensin domain) that account for 27% of the top predictions. Proteins that can be considered as TOLIPs include kazal protease inhibitors, metallothioneins, WAP proteins and several uncharacterized Epidermal growth factor (EGF) like proteins. An additional 304 sequences were scored at a moderate confidence level (P1, See Materials and Methods). A similar composition of domains was detected among these 304 moderate scored sequences. In addition, domains of “proline rich proteins” and “tripartite motif-containing proteins” were also found among the positively predicted list. The fraction of InterPro annotated proteins is indicated. Representatives of three-finger (TFP) fold proteins are distributed in all levels of positive predictions (P1–P3, see Materials and Methods).

UniProtKB compiled all mouse transcriptome under the “complete proteome” annotation (42,901 sequences). The family and domain information for the mouse proteome is quite complete, with 83% of the sequences associated with at least one InterPro term [25]. However, from a set of 7337 sequences that are shorter than 120 amino acids, only 51% of them are associated with some InterPro annotations. This observation argues that among short proteins, many are uncharacterized.

Applying ClanTox to the mouse transcriptome turned out to be a successful procedure for identifying uncharacterized TOLIPs. The ClanTox positive prediction list includes only 6% of the input (*i.e.*, all mouse short proteins); among them, 1.5% are associated with top scoring predictions (P2–P3, see Materials and Methods). Results from the ClanTox predictions according to their confidence levels for short proteins are shown (Figure 1). Interestingly, the majority of the top-ranked predictions contained signal peptide sequences (SPs) at the *N*'-termini of the sequences, indicating the secreted nature of TOLIPs. A more extensive collection of the mouse transcriptome was selected from the FANTOM mouse transcriptome annotation project [26], and about ~60 uncharacterized short sequences were identified, which complemented the TOLIP candidate list.

**Figure 2:**
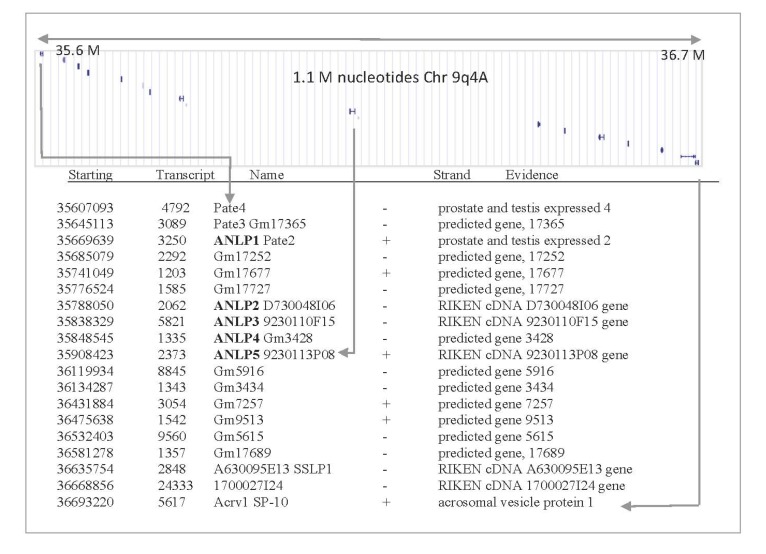
Gene organization in mouse chromosome, 9qA4. A chromosomal region that covers 1.1 million nucleotides (position 35.6–36.7 M) is shown. This region covers testis related genes and previously uncharacterized short proteins. Applying structural prediction methods indicates that this genomic segment is rich with TOLIPs that belong to the TFP fold. The chromosomal arrangement is in accord with a recent duplication event. The information for each putative gene is summarized as follows: starting nucleotide: 35607093; transcript length: 4792 nucleotide; gene name: Pate4; strand orientation: -; detailed name: prostate and testis expressed 4.

Among the proteins listed as ClanTox positive predictions (Figure 1), we focused on a subset of five uncharacterized genes that surprisingly were localized at a similar chromosomal position. The genes were named mANLP 1–5 (for mouse α-neurotoxin-like proteins). The mouse ANLPs are clustered on chromosome 9qA4 (Figure 2). The proteins in this chromosomal segment belong to the TFP fold and, specifically, to the Ly6/uPAR family. Interestingly, most of the listed genes in this chromosomal location are composed of three exons, with the first exon codes for the SP segment. Once we identify (using ClanTox) the ANLP sequences as TOLIP candidates, we apply state-of-the-art bioinformatics and genomics annotation tools to expand the list. The syntenic region in humans is located at chromosome 11q24.2 (spanning only 0.25 million nucleotides). Most genes in the cluster have a predicted cleavable SP. Several of the expected open reading frame sequences (ORFs) in this cluster lack SPs (PATE-4, D730048I06Rik and Gm17689); their expression as secretory proteins remains questionable.

### 2.2. ANLPs Belong to an Expanded Gene Family with a Single Ly6/uPAR Domain

**Figure 3:**
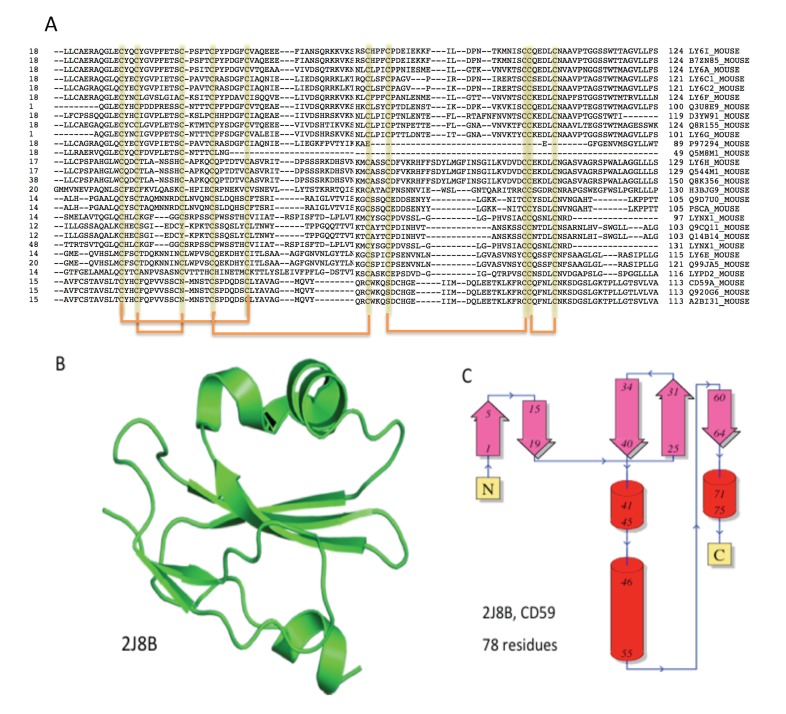
The Ly-6/uPAR/α-neurotoxin domain. (**A**) Multiple sequence alignment of 28 proteins that contain a single LU domain from mouse. All Ly-6 related proteins are localized within 0.9 million nucleotides from chromosome 15 (position 74.7 M–75.6 M). The 10 conserved cysteines that form five-disulfide bridges are marked (below the sequences); (**B**) A representative fold for the Ly-6/uPAR/α-neurotoxin is the 3D structure of human CD59. This fold is a prototype for listed proteins of the LU domain; (**C**) The secondary structure organization of 78 amino acids of the human CD59 is shown. A similar organization of β-sheets and short α-helices is found in other proteins of the LU domain.

All proteins in the mouse chromosome 9qA4 (addressed as the ANLP cluster, Figure 2) belong to the structural fold of TFP and, specifically, to the sequence based domain of the Ly-6/uPAR family (abbreviated, LU domain). ANLPs belong to the low molecular weight proteins of the LU domain family. The prototype of the family is the receptor of the Urokinase-type plasminogen activator (uPAR) that is composed of three repeated units of the LU domain. Each repeat comprises a compact fold of 80–90 amino acids. Each domain contains ten conserved cysteines (Figure 3A). Many of the LU domain family proteins are linked to the cell surface via post-translational addition of glycosylphosphatidylinositol (GPI) at their *C*'-terminal.

Figure 3 shows the multiple sequence alignment of the 28 mouse proteins that contain a single LU domain. Most of the genes are located in chromosome 15 (position 74.7M–75.6M). Note that the Ly-6 chromosomal locus includes, in addition to the numerous Ly-6 variants, proteins that share the LU fold, such as PSCA (prostate stem cell antigen), SLURP1, LYPD2 and LYNX1. Despite their strong structural resemblance (Figure 3B,C), the LU domain proteins span a wide range of activities.

### 2.3. Domain Composition and Structural View on Snake Toxins and TOLIPs

**Figure 4:**
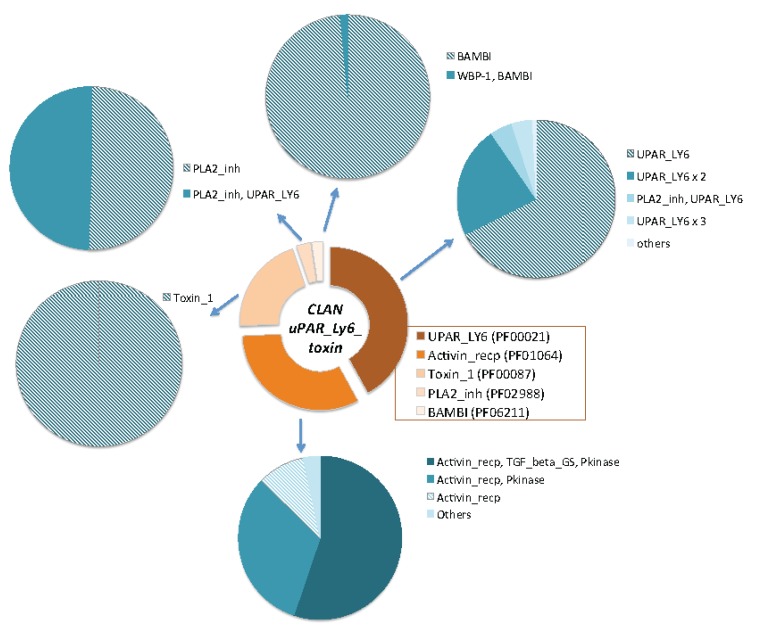
Domains’ composition of the uPAR/Ly6/CD59/snake toxin-receptor clan. The relative size of each domain family is shown in the central ring. The five domain families that compose the uPAR/Ly6/CD59/snake toxin-receptor superfamily are: (i) Toxin_1, which are proteins from elapid toxins, (ii) activin receptor, (iii) LU domain, (iv) PLA2_Inh and (v) BAMB1. The clan covers over 2900 proteins from 270 organisms. The pie charts display the compositions of the family domains for each of the five families in the clan. Proteins that are single domain are marked by a dashed pattern. All proteins of the Toxin_1 family are single domains. Note that a large fraction of the proteins in the UPAR_LY6, PF00021) are composed of two occurrences of the domain (UPAR_LY6 × 2) or a combination of the PLA2_Inh and UPAR_LY6.

The Pfam database is a large collection of protein families. Pfam clans represent the relatedness among protein families, where each clan unifies at least two domain families [27]. Figure 4 shows the relative size of each of the domain families that compose the uPAR/Ly6/CD59/snake toxin-receptor clan (CL0117). This clan includes organisms throughout the phylogenetic tree. The largest domain family in this clan is the UPAR_LY6 (PF00021). The family of Toxin_1 composes of elapid toxins. These snake toxins (Pfam family Toxin_1) are mostly short proteins with a single domain. Interestingly, PLA2_Inh (Phospholipase A2 inhibitor [28]) is an additional family that belongs to the same clan (CL0117). Many PLA2 are active components in venoms. The fifth domain family in this clan is the BAMB1 (BMP and activin membrane-bound inhibitor *N*'-terminal domain).

While in Eukaryotes, most proteins are multi-domain, all proteins that belong to Toxin_1 have only a single domain (dashed pattern). This simple composition dominates the uPAR/Ly6/CD59/snake toxin-receptor clan. Note that the proteins in four out of the five families of the clan are single domains. A rich domain composition often belongs to longer proteins. However, even these proteins are composed of a combination of domains that belong to the clan. For example, a large fraction of the proteins in the LU family (UPAR_LY6, PF00021) are composed of two appearances of the domain (UPAR_LY6 x 2) or a combination of the PLA2_Inh and UPAR_LY6). The mouse CD177 antigen (817 residues long) is composed of five repeats of the UPAR_LY6 domain. Only the family of Activin_Recp (PF01064, Figure 4) within the discussed clan includes proteins with a complex protein composition (e.g., protein kinase, TGF_beta, Figure 4). The LU domain in Activin_Recp proteins plays a role in recognition and in initiating a signal transduction cascade towards cell growth, differentiation, homeostasis, apoptosis and more.

Evidently, binding specificity of proteins is strongly dependent on the details of the structural fold, binding surfaces and backbone flexibility. In this view, we focus only on the single domain proteins from the uPAR/Ly6/CD59/snake toxin-receptor superfamily (Figure 4), as they represent secreted or membrane-attached TOLIPs. We matched the structure of these proteins in view of the functional diversity, mainly for the LU domain that mimics the structural fold of α-neurotoxins in a non-venomous context.

**Figure 5:**
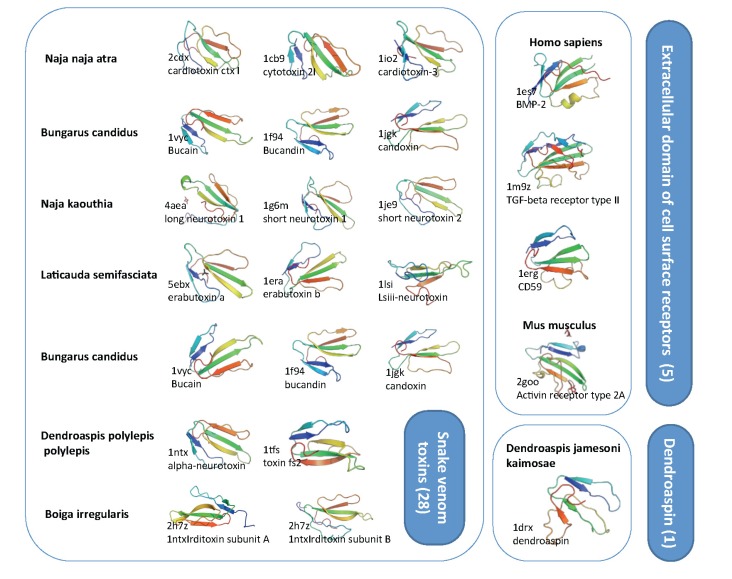
Structural view on toxin-fold (SCOP g.7.1). SCOP classification is based on SCOP 1.75B. There are three families that compose the superfamily g.7.1 including: (i) g.7.1.1, snake venom toxins (28 protein domains) (ii) g.7.1.2, dendroaspin (one protein) and (iii) g.7.1.3, Extracellular domain of cell surface receptors (five protein domains). Representative examples are shown with their Protein Data Bank (PDB) identifier and the organism. The overall representatives share less than 40% sequence identity. Note that the structural similarity is high, with three loops and nearly all structure dominated by the β-strands. The length of most of these structural domains ranges from 65–85 amino acids.

Figure 5 illustrates the superfamily that overlaps with TFPs and the sequence-based clan (CL0117, Figure 3), according to SCOP (structural classification of proteins) [29]. The superfamily from SCOP divides all the structurally solved proteins into 34 representative structures, and among them, 28 are from snake venom toxins, (overlapping Toxin_1 domain family, Figure 3). The other SCOP families are much smaller (Figure 5). The prototype of the family includes cell surface receptors of CD59, BMP-2, activin receptor and uPAR proteins. Interestingly, from a structural perspective (which often is more conserved than sequence), TOLIPs, like the Lynx-1, SLURP-1, belong to the Snake Toxin SCOP family rather than to the other structural families. Actually, the human Lynx-1 is more similar to Cobrotoxin1, Bucandin than to mammalian Activin-like family protein. The homology group in the CATH protein structure classification database [29] (CD59, 2.10.60.10) is represented by 14 structures (for <35% identity threshold). The superfamily belongs to the Ribbon Architecture.

### 2.4. Ample Binding Specificities for the Snake-Toxin-like Fold

Ly-6 molecules were initially identified in mice as lymphocyte differentiation antigens [30]. Their role in T-cell activation and lymphoblast transformation suggests that the LU domain is active in cell signaling with respect to immune response.

The first mammalian member of the Ly-6 family that is secreted is called SLURP-1. SLURP1 and Lynx1 are the best-studied examples for endogenous α-neurotoxins-like proteins [31]. We found that the identified uncharacterized TOLIPs expose evolutionary expansion events. Inspecting the TOLIPs through a chromosomal view identified an expansion of the Ly-6 family to about 45 representatives in mouse and about 25 genes in human (Figure 2 and Figure 3). When natural polymorphism and alternative spliced forms are taken into account, the number of protein products is actually much larger. We will not elaborate on the level of functional diversity that is gained due to alternative spliced protein variability.

Lynx-1 was the first example of an endogenous toxin-like protein from the mouse brain. It shares sequential and structural features with other snake venom neurotoxins [18]. It was found that it expresses in samples of tissues that are subjected to nicotine regulation. The receptor is actually expressed in the male fertility system, lung, skin and cells of the immune system. We listed the cellular functions of the Ly-6 representative members. Often, these processes are the outcome of the binding variety of ion channels, ligand receptors and other cell surface proteins (Table 1). While the conservation at the structural level among the LU domain proteins is surprisingly high (Figure 4), these proteins differ substantially in function. The different members of the Ly-6 family participate in cell recognition, adhesion, apoptosis and fertilization (Table 1). The members of the LU domains were implicated mostly in adhesion regulation and in cell processes in the context of T-cell maturation and differentiation [32]. Interestingly, the expression of Ly-6 members often governs the susceptibility of the organism to bacteria, viral infection and the pathogens’ resistance capacity [33].

**Table 1:** Functional roles of a sample of Ly6/uPAR domain family members from mammals. MAC, membrane attack complex.

Gene symbol	Biological process	Reference
CD177	Neutrophil proliferation, inflammatory settings	[34]
CD59	Regulating the action of the complement MAC	[35]
GPI-HBP	Lipid uptake from high-density lipoprotein (HDL) particles	[36]
Intectin	Intestinal epithelial apoptosis	[37]
Ly-6A	Mediating cell-cell adhesion	[38]
Ly-6C	Regulating adhesion and homing of T-cells	[39]
Lynx-1	Neuronal survival and Apoptosis	[40]
Lynx2	Potentate axon outgrowth and guidance	[41]
PSCA	Neuronal development and survival	[42]
SAMP-14	Sperm-egg interactions	[43]
SLURP-1	Implicated in a skin disorder, Mal de Meleda	[44]
SLURP-2	Induced in psoriasis vulgaris	[19]

For most of the described cellular functions (Table 1), no structural information for the specificity determinants is available. However, even without detailed structural information, the Lynx1-2, α-bungarotoxin and ANLP (not shown) act through activation and repression of the nicotinic receptors (nAChR) pathway. At the organism level, the functionality of cognitive deficits, such as schizophrenia, ADHD (Attention deficit hyperactivity disorder) and Alzheimer’s disease, was attributed to the function on the nicotinic signaling. Therefore, the cellular outcomes, such as neuronal survival or degeneration, are sensitive to the specific nature of nAChR’ modulators. In this view, the ANLPs, Lynx proteins and other TOLIPs have the potential to govern complex behavioral outcomes. For example, Lynx2 was suggested as an important component of the molecular mechanisms that control anxiety [41]. Altered glutamatergic signaling in the prefrontal cortex of Lynx2 knockout mice contributes to increased anxiety-related behaviors.

In Drosophila, Ly-6 proteins were associated with the rhythmic cycle. A screen for a sleepless gene identified a GPI anchor Ly-6-like protein, called SSS. Mutation in the genes resulted in severe effects on behaviors and longevity [45]. It was further demonstrated that SSS accelerated the kinetics of Shaker K^+^ channel currents through a direct interaction [46]. The regulation of neuronal excitability by endogenous toxin-like molecules is thus a general phenomenon of Ly-6 related proteins, as shown for Lynx, SLURP, ANLP and SSS.

### 2.5. TOLIPs Alter the Signaling Pathways of the Immune System

The Ly-6 proteins in human and mouse proteins are glycosylated and often organized as larger GPI anchored complexes (e.g., Lynx1, Lynx2, Ly6H) or secreted proteins (e.g., SLURP-1 and SLURP-2) [47]. There are 60 Ly-6-like proteins in mouse and 48 in human. Numerous Ly-6 proteins function in the context of the immune response (Table 1). Unfortunately, information on the biochemical and direct interactions of some of the Ly-6 proteins is still fragmentary. Most hematopoietic cells (e.g., B-, T-, NK-, monocytes, neutrophils, dendritic cells) express one or more members of the Ly-6 superfamily. Ly-6 proteins that are expressed on the membrane of the lymphoid and myeloid cells are suspected in adhesion and cell signaling. Interestingly, a region in chromosome 15 that covers the Ly-6 complex region (Figure 3A) was also found to overlap a dominant factor for virus susceptibility [33].

The keratinocytes participate in the first-line of defense against pathogens. Members of the Ly-6 proteins were implicated in the skin cell function and the innate immune system. The expression of a SLURP-2 is upregulated in lesions of psoriasis vulgaris. The pathology of psoriasis is manifested by the hyperproliferation of keratinocytes and T-cell induced inflammation. Thus, SLURP-2 plays a role in the pathogenesis of psoriasis vulgaris by interfering with the immunological network of the skin [48]. The close homologue, SLURP-1, directly binds α7 nAChR in keratinocytes.

Snake α-bungarotoxin, Lynx, SLURP and ANLP bind directly to nAChR receptor (albeit of different sub-types). However, the molecular binding specificity of the entire repertoire of Ly-6 proteins is beyond the direct effect on nAChRs. For example, the Ly6G reduced the ability of neutrophils to respond to external stimuli. This is performed through a direct binding between Ly6G with β2 (CD11a and CD11b) integrins. Thus, Ly6G controls adhesion molecules and, consequently, the recruitment capabilities of neutrophils [49].

CD59 is an important regulator of the immune system via its role on complement complex formation. Cells that express CD59 inhibit the complement complex formation and prevent self-attack [50]. CD59 is a short (128 amino acids), globular GPI-anchored glycoprotein. Its main function is inhibition of the complement membrane attack complex (MAC). The binding surfaces of CD59 were determined from its 3D structure. It was shown to bind C8 and C9 during MAC assembly [50]. The binding capacity of CD59 is not limited to the complement subunits. An additional physiological ligand of CD59 on T-cell membranes was identified as the CD2. The protein is concentrated in lipid rafts and acts in signaling through T-cell adhesion and activation. Functions, such as neutrophil activation and apoptosis, have also been attributed to CD59 [51].

### 2.6. TOLIPs Linkage to Pathologies and Diseases

**Figure 6:**
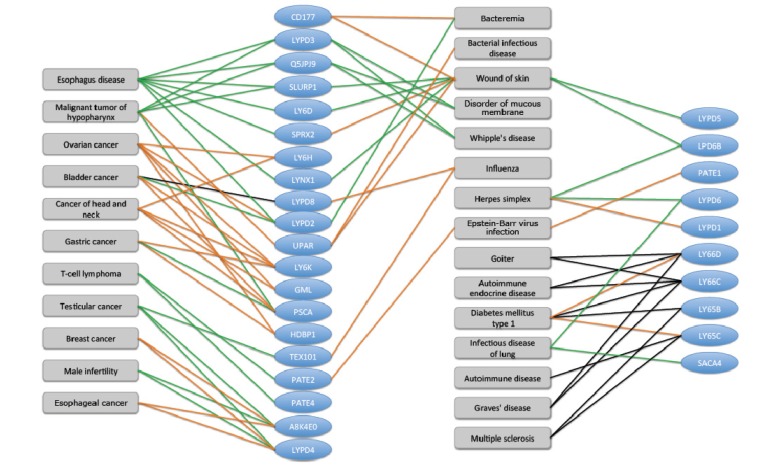
Human Ly-6 proteins and their disease associations. The most significant 30 human uPAR/Ly6 (LU) genes that were implicated in disease and pathologies are shown (blue ovals). There are 48 human LU family members. Data on disease-gene association was extracted from the UniProtKB cross reference of NextBio. Only high score (score > 0.8) disease-gene associations supported by at least two genes from the LU domain family are reported. The relative expression level of LU domain proteins in the context of diseases is colored to indicate change in the gene expression. The induction and suppression are colored red and green, respectively. Several disease associations are based on genomics screening and GWAS (genome-wide association studies, black edges). We noted diseases such as “Wound of the skin” and “Diabetes type I” as “hub” connecting dominating genes. These diseases are the manifestation of an imbalance of the immune system.

Cell surface proteins are often associated with cancer diagnosis’ markers. Numerous such clinical markers are GPI-anchored cell-surface proteins that belong to the Ly-6/uPAR family. In this view, some Ly-6 proteins were implicated as potential therapeutic drugs [52]. The significant biological association of the LU domain human proteins as markers for a large number of cancers, as well as to immunological-based pathologies, is shown in Figure 6.

Examples for a clinical relevance of these proteins are illustrated for PSCA and CD59. PSCA is a GPI-anchored protein expressed on immature lymphocytes. The activin-type receptor domain in PSCA suggests that binding to TGF-β may activate its signaling pathway. The protein is expressed in the normal human prostate, but is overexpressed in prostate cancers [53]. Moreover, the PSCA antibody has recently been reported to have anti-tumor activity in preclinical models.

The role of CD59 in complex diseases has only recently been proposed. A genetic basis for a life-threatening disease, named paroxysmal nocturnal hemoglobinuria (PNH, [54]), was associated with a failure of CD59 to attach to the membrane by the GPI anchor. A similar pathology, called chronic inflammatory demyelinating polyradiculoneuropathy (CIDP), was shown to be a result of a single mutation in CD59 itself [55].

## 3. Experimental Section

### 3.1. Data Collection

UniProtKB [56] was used as an annotation source for “Signal peptide” and “InterPro” [25]. InterPro unifies the current knowledge on domains and families from many structure- and sequence-based resources. Sequences marked as “fragments” by UniProtKB were excluded. The FASTA file from the short set of mouse proteome from UniProtKB was used as input for ClanTox prediction [23]. About 700 FANTOM 4.0 [26] sequences that encode proteins shorter than 120 amino acids were also used as ClanTox input.

### 3.2. ClanTox Prediction

ClanTox is available as a server [5]. The input to ClanTox is a list of FASTA sequences (up to 10,000 in a single operation). The results are a list of predictions that are marked at 4 confidence levels: positive (P1, P2, P3 for moderate, high and very high, respectively) and negative predictions (N). The performance of ClanTox in term of prediction accuracy was reported [23]. Interestingly, even at the moderate prediction level (P1), many of the candidates are valid for functional assessment and are genuine TOLIPs. False positives from ClanTox results include mainly the keratins, Zn-finger and RNAse-like domains and, to a lesser extent, also, proteins with a plexin/ semaphorin domain.

### 3.3. Bioinformatics Analysis Tools

SignalP 4.0 was applied for prediction of signal peptides [57]. HHpred was used to identify remote homologues [58]. The platform supports ClustalW2 and Cobalt multiple alignment. Alignment viewer tools were used at default parameters. HHpred is a sensitive algorithm that is based on Hidden Markov model (HMM) profiles comparisons for proposing the most likely structure of domain family assignments. We applied HHpred to build an HMM from the query sequence and compared it with a library of HMMs representing all known 3D-structures from the PDB. Structural classifications used are according to SCOP and CATH [29].

## 4. Conclusions

This research presented a platform for the discovery of TOLIPs from short and uncharacterized mouse proteome. The TOLIPs that we investigated resembles animal neurotoxins (e.g. cobrotoxin1, bungarotoxin, bucandin) that belong to the clan of Ly6/uPAR/toxin/activin receptor. Many of these proteins are related to cancer diagnosis and act as markers for immunological cells. We conclude that the innate immune system is a preferred site of action for these toxin-like proteins. We anticipate that the modulatory role on cell signaling by endogenous toxin-like proteins is evolved to meet the cellular complexity of the immune system in mammals.

While most animal toxins cause an irreversible inhibition on ion channels, the discussed endogenous mammalian TOLIPs that belong to the TFP superfamily act not only to modulate receptors in the brain, but probably also in a wide range of cellular function, such as adhesion and cell migration. The TOLIPs (from human and mouse) present a novel mode of reversible regulation by protein-interaction to cell surface molecules. The role of TOLIPs in communicating conditions of immunological and neurological imbalance remains an exciting research area.

## Abbreviations

ADHD: attention deficit hyperactivity disorder
ANLP: α-neurotoxin-like protein
ClanTox: classifier of animal toxins
GPI: glycosylphosphatidylinositol
GWAS: genome-wide association studies
LPS: lipopolysaccharide
MAC: membrane attack complex
MS: mass spectrometry
nAChR: nicotinic acetylcholine receptor
OCLP: omega conotoxin-like protein
PSCA: prostate stem cell antigen
SP: signal peptide
TFP: three-finger proteins
TNF: tumor necrosis factor
TOLIP: toxin-like proteins
uPAR: urokinase-type plasminogen activator
